# Access to information in school and the use of psychoactive substances in Brazilian students – A multilevel study

**DOI:** 10.1016/j.abrep.2018.07.004

**Published:** 2018-07-29

**Authors:** Cristine Scattolin Andersen, Rogério Lessa Horta, Marcos Pascoal Pattussi

**Affiliations:** a Programa de Pós Graduação em Saúde Coletiva, Universidade do Vale do Rio dos Sinos (UNISINOS), Av. Unisinos, n° 950 – Cristo Rei, São Leopoldo, RS CEP 93020-190, Brazil; b Núcleo de Saúde e Segurança do Trabalho, Instituto Federal de Educação, Ciência e Tecnologia Farroupilha (IFFar), Rua Esmeralda, 430 – Faixa Nova de Camobi, Santa Maria, RS CEP 97110-767, Brazil

**Keywords:** Alcohol, Tobacco, Adolescents, Education, Multilevel modelling

## Abstract

**Introduction:**

Use of tobacco, alcohol and other drugs can be considered a global health problem, which typically begins in adolescence. Unsupervised access to information may arouse the adolescent's interest and predispose the use of drugs.

**Methodology:**

This is a cross-sectional study using data from National School-based Health Survey (PeNSE, 2012), with sample of 109,104 Brazilian students in 42.717 schools. Outcomes were: self-reported use of alcohol, tobacco, and other drugs in the past 30 days. Main exposures were contextual and included: library and media resources availability, computer room and internet available at school. Data analysis included multilevel logistic regression.

**Results:**

Prevalence of alcohol use was 25.2% (IC95% 24.7–25.6), tobacco use was 5.3% (IC95% 5.1–5.5) and use of other drugs was 2.6% (IC95% 2.5–2.7). Multilevel analysis showed that recent use of alcohol and tobacco was associated to the presence of computer room and internet, while the use of other drugs presented an association with all media.

**Conclusion:**

Results indicate that supervision in access to information and communication resources may play a role on the prevention of alcohol, tobacco and other drugs use by students.

## Highlights

What do we already know about this?
• The use of drugs by students receives intrinsic and extrinsic influences. - State and parental control seems to be effective in preventing initiation and maintenance of this consumption.
What does this study add?
• Supervision of online adolescent behavior by adults may play a role on the prevention of the use of psychoative drugs.

## 1 Introduction

The use of tobacco, alcohol and other drugs among students can be considered a global public health with high prevalence worldwide (Carlini et al., 2010; Hibell et al., 2007). The use generally begins in adolescence (Hodder et al., 2011; Lovato et al., 2013), an age group in which the vulnerability to social peer pressure tends to be stronger (Franelić, Kuzman, Šimetin, & Kern, 2011; Scull, Kupersmidt, Parker, Elmore, & Benson, 2010).

School environment can be considered an ideal place to health promotion actions that include the prevention of substance use (Organizacion Panamericana de la Salud, 1995; World Health Organization, 2009) because the wealth of knowledge and both formal (classes and lectures) and informal (daily life) information provided (Hardoff, Stoffman, & Ziv, 2013; Kuntsche & Jordan, 2006; Pavani, Silva, & Moraes, 2009).

On the other hand, being a place of social gatherings and ties, it may also be a conducive environment that may favour the start and maintenance of drug use in adolescents (Backes et al., 2014; World Health Organization, 2009). Access to information may contribute to increase the drug use prevalence (van der Meer, Oliveira, Ribeiro, & Nappo, 2010). Network of peers may propitiate substances consumption by the diffusion of positive experiences, dissemination of means of access and propagation of alcohol and other drugs use (Scull et al., 2010). The inquisitiveness and risk taking behaviour characteristic of school age children (Eisenstein, 2005; van der Meer et al., 2010), together with permissive family factors (Brooks, Magnusson, Spencer, & Morgan, 2012; Calafat, García, Juan, Becoña, & Fernández-Hermida, 2014), and the possible unsupervised information broadcast may, in opposition to whats is desired, arouse adolescent's interest and stimulate the use of alcohol, tobbacco and/or drugs (Kuntsche & Jordan, 2006; van der Meer et al., 2010).

Because the available evidence seems to suggest that information access may both prevent or predispose the use of psychoactive substances by students (Hardoff et al., 2013; van der Meer et al., 2010) the objective of the presente study was to analyze the association between the information and communication structure of the school and the use of psychoactive substances in the preceding 30 days by Brazilian students.

## 2 Methodology

This article uses data from the National School-based Health Survey (PeNSE) 2012 (Instituto Brasileiro de Geografia e Estatística, 2012), a cross-sectional, triennial study, performed for the first time in 2009, carried out by the Brazilian Institute of Geography and Statistics (IBGE) in partnership with the Ministry of Health, with the support of the Ministry of Education of Brazil.

Sampling plan was comprised of 27 geographic strata corresponding to all capitals and the Federal District. The remaining cities were grouped within each of the five geographic regions of the country (North, Northeast, South, Southeast and Center-West) forming five geographic strata. The sample of each geographic stratum was allocated proportionally to the number of schools according to the administrative dependency. Schools with <15 students in the desired grade were excluded (9th grade from elementary school). For each stratum, a conglomerate sample was selected in two stages: schools and classrooms. In the strata formed by cities that were not capitals the primary sampling units were the groups of cities, the secondary units were schools and the classrooms of these schools were the tertiary sampling units.

The final sample was representative of the country, the 27 capitals and the Federal District, all these capitals and each of the five major geographic regions.

The IBGE teams visited the selected classes in each school and all the students from these classes present on the day were invited to participate in the research. The students answered an electronic questionnaire through a smartphone, which had the free and informed consent form on the first screen. Managers or school representatives were interviewed about the characteristics of the school.

From the PeNSE-2012 database, individual and contextual variables were selected. The outcomes were: alcohol, tobacco and other drugs use in the preceding 30 days (use of at least one of the following substances: cannabis, cocaine, crack, glue, *loló*, *lança perfume*, ecstasy, oxy, substances currently considered illegal in Brazil).

The main exposures were contextual and corresponded to the information and communication resources including availability of: library, media/communication facilities, computer room and internet access for students in schools. Such information was obtained by the response of the manager or the responsible for the school, in yes/no items.

Individual confounding factors included: age in years (Carlini et al., 2010; Hibell et al., 2007), male or female (Carlini et al., 2010; Hibell et al., 2007), maternal schooling (Malta, Mascarenhas, Porto, Barreto, & Neto, 2014), school's administration (public or private) (Carlini et al., 2010), student engaged in a remunerated activity (Souza & Silveira-Filho, 2007), reported having had sexual relations (Malta et al., 2014), live with parents at the time of the interview (Brooks et al., 2012; Horta, Horta, & Pinheiro, 2006), students' perception of how parents would react if they come home drunk (Brooks et al., 2012; Larrosa & Palomo, 2010), reported having suffered domestic violence in the preceding 30 days (Larrosa & Palomo, 2010), reported having been victim of bullying in the preceding 30 days (Luk, Wang, & Simons-Morton, 2012; Strauch, Pinheiro, Silva, & Horta, 2009), reported missing school without parents' permission in the preceding 30 days (Fraile Duvicq, Pereira, & Carvalho, 2004; Malta et al., 2014), reported having close friends (Fazel, Hoagwood, Stephan, & Ford, 2014a; Fazel, Patel, Thomas, & Tol, 2014b).

Preliminary, descriptive and univariate analyses were performed in the SPSS 22 program using Pearson's Chi-Square (χ^2^) or linear trend test. The multivariate analysis used multilevel logistic regression to obtain crude and adjusted odds ratios (OR) through the MLWin program 2.35. The empty model showed significant contextual variance for the three outcomes, justifying the continuation of the analyses by multilevel technique. Considering the large sample size and greater probability of type 1 error, only the individual independent variables associated with outcomes at a significance level of <1% (*p* < 0,01) in the univariate analysis were included in the final model. Similarly to other studies (Agaku, Caixeta, de Souza, Blanco, & Hennis, 2016; Organizacion Panamericana de la Salud, 1995), the race variable was not associated with the outcomes and therefore, was not included in the final model. Age and sex-stratified analyses were also performed for the associations studied, but no important differences were found and thus these data were not reported.

## 3 Results

In total, the sample consisted of 109,104 morning-class students enrolled in the 9th grade of 42,717 Brazilian schools. Among these, 25.2% (IC95% 24.7–25.6) reported consumption of alcohol in the preceding 30 days, while 5.3% (IC95% 5.1–5.5) smoked and 2.6% (IC95% 2.5–2.7) used other drugs in the same period. Most of the students were female, not white, in the expected age for the grade and studied in public schools. Higher prevalence of the substances (alcohol, tobacco and other drugs) were verified in the interviewees reporting that the family would not care if they came home drunk. Table 1 shows the sample distribution and the behaviour of the outcomes according to individual confounding factors.

**Table 1:** Sample distribution and prevalence of outcomes according to individual confounding factors (*n* = 109,104).

Variables	n	Alcohol use in 30 days		Tobacco use in 30 days		Illicit drugs use in 30 days	
		%	IC95%	%	IC95%	%	IC95%
Gender							
Male	52,015	24.1	23.8–24.5	5.7	5.5–5.9	3.2	3.1–3.4
Female	57,089	26.1	25.7–26.4	4.9	4.6–5.1	2.1	1.9–2.2
Age (years)							
13 or less	22,443	17.5	17.0–18.0	2.7	2.5–2.9	1.3	1.1–1.4
14 or 15*	72,005	25.0	24.7–25.3	5.0	4.8–5.1	2.5	2.4–2.6
16 or more	14,656	37.7	36.7–38.4	10.7	10.2–11.2	5.1	4.8–5.5
Race/colour							
White	37,674	25.3	24.9–25.6	4.9	4.7–5.2	2.7	2.6–2.9
Non white	71,430	25.1	24.7–25.4	5.5	5.3–5.6	2.6	2.4–2.7
Geographic region							
North	22,774	21.0	20.5–21.5	4.9	4.6–5.2	1.7	1.5–1.8
Northeast	31,301	22.2	21.8–22.7	3.7	3.4–3.9	1.8	1.6–2.0
Southeast	19,660	26.4	25.8–27.0	5.0	4.7–5.3	3.2	3.0–3.4
South	14,878	32.2	31.4–32.9	7.9	7.4–8.3	4.4	4.1–4.7
Centre-West	20,491	28.0	27.4–28.6	6.6	6.2–6.9	3.0	2.8–3.3
Administrative dependence							
Private	86,600	22.5	22.0–23.0	3.4	3.2–3.7	2.4	2.2–2.6
Public	22,504	25.8	25.6–26.1	5.8	5.6–5.9	2.7	2.6–2.8
Remunerated activity							
Yes	13,013	37.8	37.0–38.6	10.3	9.8–10.8	5.7	5.3–6.1
No	96,091	23.5	23.2–23.7	4.6	4.5–4.7	2.2	2.1–2.3
Maternal Schooling							
University	59,386	24.8	24.1–25.4	4.5	4.3–4.8	3.0	2.7–3.2
High school	30,353	24.9	24.4–25.4	5.1	4.9–5.4	2.7	2.6–2.9
Elementary school	19,042	25.4	25.1–25.8	5.6	5.4–5.8	2.4	2.3–2.5
Family structure							
Lives with both parents	64,304	22.5	22.1–22.8	4.2	4.0–4.3	2.1	2.0–2.2
Lives with one parent	37,541	28.8	28.3–29.3	6.7	6.4–6.9	3.3	3.2–3.5
Does not live with either parent	7065	30.3	29.3–31.4	8.0	7.4–8.6	3.5	3.1–3.9
Suffered familial violence in the last 30 days							
Yes	11,470	40.6	39.7–41.5	12.8	12.2–13.4	7.1	6.6–7.6
No	97,010	23.3	23.1–23.6	4.4	4.2–4.5	2.1	2.0–2.2
Missed school with no permission in the last 30 days							
Yes	25,843	38.7	38.1–39.3	11.2	10.9–11.6	5.6	5.3–5.9
No	82,971	21.0	20.7–21.2	3.4	3.3–3.5	1.7	1.6–1.8
Parents' reaction if student come home drunk							
Care very much	97,085	21.7	21.4–22.0	3.9	3.8–4.0	1.7	16–1.8
Do not care much	6708	59.8	58.6–61.0	14.7	13.9–15.6	9.1	8.4–9.8
Do not care	1943	60.6	58.4–62.9	26.8	24.8–28.8	16.3	14.6–17.9
Do not know	2885	39.8	38.0–41.7	14.3	13.0–15.6	8.9	7.8–9.9
Victim of bullying in the last 30 days							
Never	71,705	23.8	23.4–24.1	4,8	4.7–5.0	2.4	2.3–2.5
Rarely/Sometimes	29,605	27.7	27.2–28.2	5,7	5.5–6.0	2.9	2.7–3.1
Most of the time/Always	7193	27.8	26.8–28.9	7,6	6.9–8.2	3.5	3.1–4.0
Reported having close friends							
>3	7401	25.0	24.7–25.3	5,0	4.8–5.1	2.4	2.3–2.5
One or two	17,322	26.2	25.5–26.8	6,1	5.7–6.4	3.1	2.8–3.3
None	3661	23.3	22.0–24.7	7,7	6.8–8.5	4.2	3.6–4.9
Sexually active							
No	75,882	17.1	16.8–17.3	2,1	2.0–2.2	0.8	0.7–0.8
Yes	32,835	44.1	43.5–44.6	12,7	12.3–13.0	6.9	6.6–7.2

*Expected age for the grade.

Most Brazilian schools had information and communication resources, and the highest prevalence of alcohol, tobacco and other drugs was found where these resources were available (Table 2).

**Table 2:** Sample distribution and prevalence of outcomes according to contextual exposures (n students = 109,104, n schools = 42,717).

Variable	n students	n schools	%	IC95%
Alcohol use in the last 30 days				
Library				
Yes	98,130	98,130	25.2	25.0–25.5
No	10,707	10,707	24.5	23.6–25.3
Media resources				
Yes	69,466	69,466	25.3	25.0–25.6
No	39,371	39,371	25.0	24.5–25.3
Computer room				
Yes	95,564	95,564	**25.4**	**25.1–25.6**
No	13,273	13,273	**23.8**	**23.1–24.6**
Internet available				
Yes	83,449	83,449	**25.6**	**25.3–25.9**
No	25,655	25,655 (23.5)	**23.6**	**23.0–24.1**

Tobacco use in the last 30 days				
Library				
Yes	98,130	98,130 (90.2)	**5.4**	**5.1–5.5**
No	10,707	10,707 (9.8)	**4.3**	**3.9–4.7**
Media resources				
Yes	69,466	69,466 (63.8)	5.4	5.2–5.6
No	39,371	39,371 (36.2)	5.1	4.9–5.3
Computer room				
Yes	95,564	95,564 (87.8)	**5.4**	**5.2–5.5**
No	13,273	13,273 (12.2)	**4.7**	**4.4–5.1**
Internet available				
Yes	83,449	83,449 (76.5)	**5.4**	**5.5–5.6**
No	25,655	25,655 (23.5)	**4.9**	**4.6–5.2**

Other drugs use in the last 30 days				
Library				
Yes	98,130	98,130 (90.2)	**2.7**	**2.6–2.8**
No	10,707	10,707 (9.8)	**2.0**	**1.7–2.2**
Media resources				
Yes	69,466	69,466 (63.8)	**2.8**	**2.7–2.9**
No	39,371	39,371 (36.2)	**2.3**	**2.2–2.4**
Computer room				
Yes	95,564	95,564 (87.8)	**2.7**	**2.6–2.8**
No	13,273	13,273 (12.2)	**2.1**	**1.9–2.4**
Internet available				
Yes	83,449	83,449 (76.5)	**2.7**	**26–2.8**
No	25,655	25,655 (23.5)	**2.3**	**2.1–2.5**

Note: bold figures were statistically significant at the 1% level (p < 0,01).

In the multivariate analysis, alcohol and tobacco use were strongly associated to presence of fast media (computer/internet room). On the other hand, other drugs use in the last month was associated to the presence of all types of media (Table 3).

**Table 3:** Crude and adjusted odds ratios obtained through multilevel logistic regression for use in the last 30 days of alcohol, tobacco and other drugs according to the type of media present at the school (n students = 109,104, n schools = 42,717).

Variable	Crude OR	(IC 95%)	Adjusted OR	(IC95%)
Alcohol use in 30 days^a^				
Library	1.04	0.97–1.11	1.07	0.99–1.15
Media resources	1.02	0.98–1.07	**1.06**	**1.01–1.11**
Computer room	**1.09**	**1.02–1.17**	**1.13**	**1.06–1.21**
Internet available	**1.13**	**1.08–1.19**	**1.19**	**1.13–1.26**

Tobacco use in 30 days^b^				
Library	**1.26**	**1.09–1.45**	1.03	0.93–0.15
Media resources	**1.09**	**1.00–1.19**	**1.19**	**1.09–1.30**
Computer room	**1.17**	**1.03–1.32**	**1.19**	**1.05–1.35**
Internet available	**1.15**	**1.04–1.27**	**1.22**	**1.11–1.34**

Other drugs use in 30 days^c^				
Library	**1.44**	**1.17–1.78**	**1.54**	**1.24–1.91**
Media resources	**1.28**	**1.13–1.45**	**1.42**	**1.25–1.61**
Computer room	**1.31**	**1.08–1.58**	**1.33**	**1.10–1.62**
Internet available	**1.25**	**1.09–1.45**	**1.34**	**1.16–1.56**

Obs.: the reference categories refer to the absence of such resources in the schools. Bold figures were statistically significant at the 1% level (*p* < 0,01).

a Adjusted for sex, age, administrative dependence, remunerated activity, lives with parents, family's reaction if the interviewee drank, domestic violence, victim of bullying, misses class without permission from the parents, sexual intercourse.

b Adjusted for sex, age, adminsitrative dependece, maternal schooling, remunerated activity, lives with parents, family's reaction if the interviewee drank, domestic violence, victim of buylling, misses class without permission from the parents, report having close friends, sexual intercourse.

c Adjusted for sex, age, maternal schooling, remunerated activity, lives with parents, family's reaction if the interviewee drank, domestic violence, victim of bullying, misses class without permission from the parents, report having close friends, sexual intercourse.

## 4 Discussion

Results showed that the availability of information and communication resources in schools was associated with higher prevalence of alcohol, tobacco and other drugs use by students. More specifically, the presence of fast media, which include digital technologies such as computer rooms and internet access, was associated with the recent consumption of the psychoactive substances. Moreover, the consumption of drugs considered illicit in Brazil was also associated with the availability of slow media and access to information, such as libraries, but the same did not happen for alcohol and tobacco. Although the presence of communication/information infrastructures can be considered beneficial to prevent these behaviours (Champion, Newton, Barret, & Teesson, 2013; Kuntsche & Jordan, 2006), the current result indicates that the access to information can also be a condition associated with the use of drugs (van der Meer et al., 2010).

Factors associated with use or repeat the use of psychoactive drugs have been well documented. Family conflicts (Brooks et al., 2012; Calafat et al., 2014) and substance availability (Larrosa & Palomo, 2010) have been associated with higher use. Religiosity has also been associated with lower prevalence of use (Tavares, Béria, & Lima, 2004), whereas influence of peers may either favour or prevent the use of psychoactive substances (Fazel, Patel, et al., 2014b; Hardoff et al., 2013). Communication processes have also been related to drug use (Kuntsche & Jordan, 2006; van der Meer et al., 2010). The use of partial or not credible messages based only on negative effects of drugs and with the intention to shock, has not shown to be effective in use prevention. This is because the student is not always able to recognize the broadcasted danger or the loss control he would experience when observing drug users around (Gil, Mello, Ferriani, & Silva, 2008; van der Meer et al., 2010). The network of relationships is an important part of communication and access to information processes (Gil et al., 2008; Kuntsche & Jordan, 2006). At the same time, disclosure of the positive effects of substances may stimulate young people's curiosity and favour the initiation of or maintenance of use (Kuntsche & Jordan, 2006; van der Meer et al., 2010). The choice to a healthier decision (no consumption) in front of a risky situation (drug availability) is favoured by the development of the young's social skills, a competence that is not built by providing information and knowledge, untied from actions that involve emotional and social components (Backes et al., 2014).

The higher substance use was associated with use of virtual communication devices (Scull et al., 2010) and of social networks (Tavares et al., 2004), apparently influenced by glamor messages and lack of consequences regarding their use by adolescents (Pinsky & El Jundi, 2008; Vendrame, Pinsky, Faria, & Silva, 2009). Peer influence must be considered, since higher use of these devices seems to amplify social interaction, complementing the physical (offline) relationships (Gommans et al., 2015) and tending to amplify peer influence in relation to the use of drugs (Gommans et al., 2015; Gutierrez & Cooper, 2016). It is known that social circles are less susceptible to be controlled by public policies, but they may also be influenced by the family (Brooks et al., 2012). Families are relevant when it comes to the credibility of knowledge about drugs acquired by students from their family or from their friends' networks and this has been considered by students as appropriated as school's formal education on the subject (Gil et al., 2008; van der Meer et al., 2010).

The results of the present study should be interpreted in light of certain limitations. First, because its cross-sectional nature, the study is limited to identification of associations, and cannot establish causal relationships. Reverse causality may exist, since both exposure and outcome are measured at the same moment in time. Thus, it is not possible to specify whether the access to internet resources preceded or resulted in the drug use. For example, the fact that the student can seek access to the virtual networks (mainly fast media) because he makes use of psychoactive substances can not be ruled out. Second, data collection was performed on a single day, determining the loss of students who were absent. Drug use may be associated with school absenteeism (Silva, Pavani, Moraes, & Neto, 2006). Third, only day-class students were interviewed. Night-class students tend to report higher alcohol, tobacco and other drugs use (Huang, Lin, Lee, & Guo, 2013; Silva et al., 2006). Thus, because absent and night class students were excluded, the prevalences reported here may be underestimated. Finally, since PeNSE was not primarily designed to identify the factors associated with the use of psychoactive substances there is risk of unmeasured confounding at both school and individual levels.

Despite the fact that information and communication resources can be useful tools to aid in the education and prevention of substance use by students, such resources may also undermine this effort. Unsupervised access to these resources, especially fast media (internet), is probably has been associated with a higher prevalence of recent substance use as a way to enhance peer influence. In assessing access to information and communication resources, this study does not explore the conditions of use. There is no information, for example, if the students were supervised or not while accessing these media. On the other hand, it has already been proposed that adult supervision of adolescent activities may be associated with lower prevalence of drug use (Sigfusdottir, Kristjansson, Gudmundsdottir, & Allegrante, 2011). Therefore, it is likely that supervision of online adolescent activities would favour the use of these resources for the development of health skills and growing awareness of the student within his/her social and family networks.

The results presented here suggest that the availability of information and communication resources in schools may play a role in the use of psychoactive substances by students. This association cannot be satisfactorily explained by current knowledge. In this sense rather than closing, it opens the discussion on this subject. The use of longitudinal and/or qualitative approaches may help to better elucidate this question.

## Disclosure of interests

The authors report no conflicts of interest.

## Funding

This study uses secondary data collected with resources from the Brazilian Institute of Geography and Statistics (IBGE) in partnership with the Ministries of Health and Education.
